# Completely laparoscopic repair for recurrent inguinal hernia that developed after open posterior mesh repair

**DOI:** 10.1111/ases.12810

**Published:** 2020-06-08

**Authors:** Daisuke Morioka, Yusuke Izumisawa, Norio Ohyama, Kazuya Yamaguchi, Nobutoshi Horii, Fumio Asano, Masaru Miura, Yoshiki Sato

**Affiliations:** ^1^ Department of Surgery Yokohama Ekisaikai Hospital Yokohama Japan

**Keywords:** completely laparoscopic repair, open posterior mesh repair, recurrent inguinal hernia

## Abstract

Surgeons tend to avoid performing completely laparoscopic repair (CLR) for recurrent inguinal hernia (RIH) that developed after the open posterior mesh repair (OPMR). For many, totally extraperitoneal repair or transabdominal preperitoneal repair after OPMR seems difficult because the previously placed mesh may pose an obstacle during the exfoliation of the parietal peritoneum. Moreover, these procedures could cause chronic pain if the “trapezoid of disaster” is injured. In this small case series, we describe our operative technique for CLR for RIH after OPMR, including modified transabdominal preperitoneal repair and modified intraperitoneal onlay mesh repair. The short‐term and midterm outcomes of this procedure are also reported. Although we recognize the need for further analysis involving many more cases and a longer follow‐up period, we will continue to perform CLR for RIH after OPMR because the results of this small case series were favorable.

## 1 INTRODUCTION

According to the current treatment guidelines of the HerniaSurge Group, laparoscopic repair is recommended for treating recurrent inguinal hernia (RIH) after primary anterior open repair (PAOR).^1^ This is because the Lichtenstein patch repair (LPR) is the most common method of PAOR, especially in Europe and the United States. Also, laparoscopic repair, including totally extraperitoneal repair and transabdominal preperitoneal repair (TAPP), can be performed easily after LPR, during which the parietal peritoneum behind the posterior floor is nearly untouched. However, in Japan, LPR is less common than in Europe and the United States^2^; rather, open posterior mesh repair (OPMR), including mesh plug repair (MPR) and transinguinal preperitoneal mesh repair (TIPPMR), is the most common open repair. Therefore, completely laparoscopic repair (CLR) for RIH that develops after PAOR has been rarely reported in Japan.^3^, ^4^

After OPMR, totally extraperitoneal repair or TAPP seems difficult because the previously placed mesh may be an obstacle during the exfoliation of the parietal peritoneum. During exfoliation, these laparoscopic procedures appear likely to cause chronic pain if the “trapezoid of disaster,”^1^, ^3^, ^4^, ^5^ to which the previously placed mesh was adhered, especially in TIPPMR, is injured. For these reasons, even in Europe and the United States, surgeons tend to avoid performing CLR for RIH after OPMR for fear of patients developing chronic pain.^1^ As such, CLR for RIH after OPMR is not widely performed.^1^, ^2^, ^3^, ^4^, ^5^ However, a meta‐analysis showed that the incidence of chronic pain was similar between the open and laparoscopic approaches for RIH. Despite this, open repair for RIH is considered more likely than laparoscopic repair to cause chronic pain.^5^

We believed that open repair for RIH after OPMR is likely to injure the nerves in the scar tissue in the inguinal canal and thus more likely to cause chronic pain than CLR. Thus, we initiated a program of the CLR for RIH that developed after OPMR in April 2016, which was approved by our institutional review board approve (no. YEH‐2016‐S‐01). We herein report a small case series of CLR for RIH after OPMR.

## 2 MATERIALS AND SURGICAL TECHNIQUES

**FIGURE 1:**
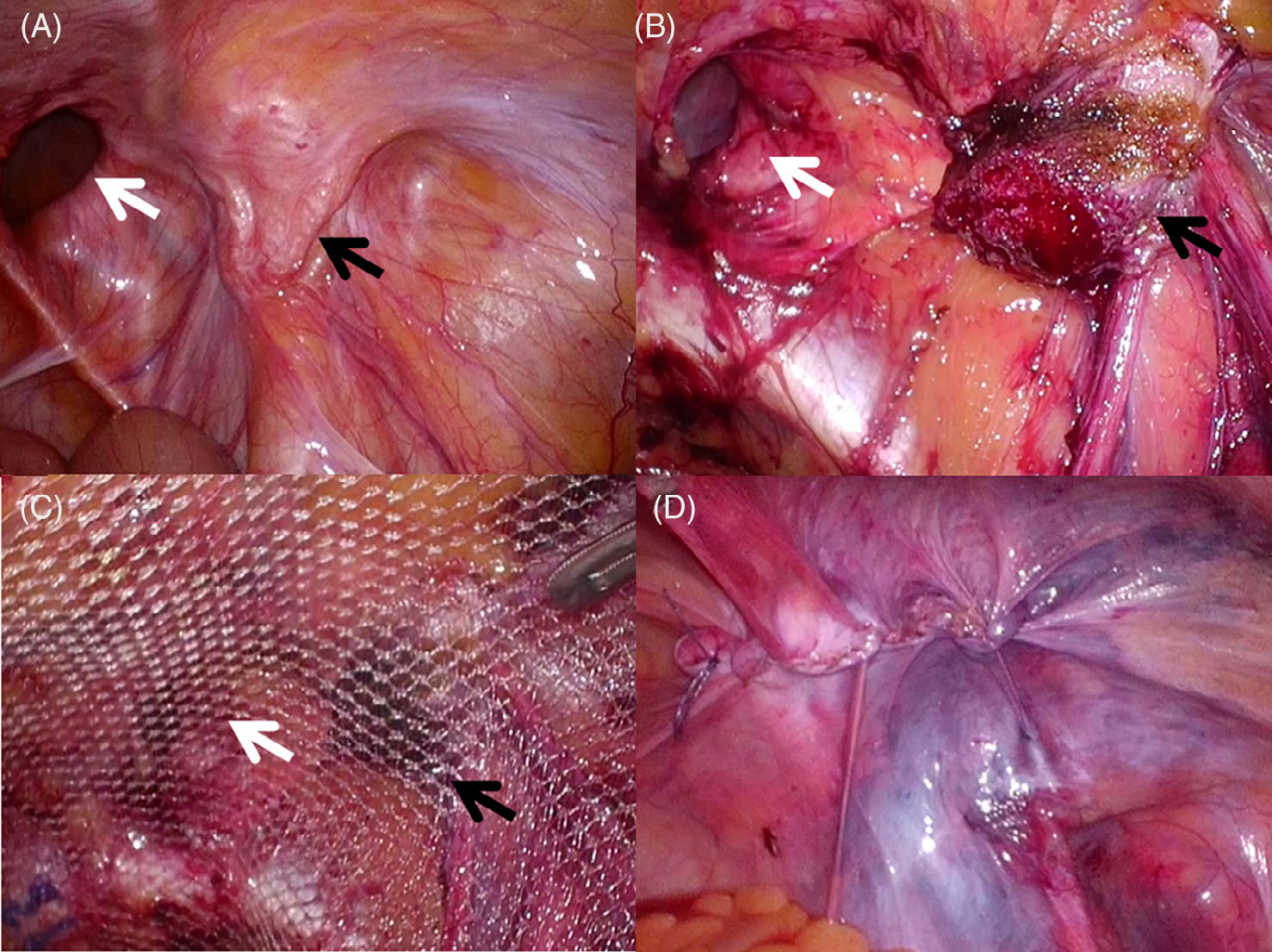
Right recurrent direct inguinal hernia that developed after mesh plug repair. (A) The hernia defect was observed distant from the previously placed mesh. (B) The mesh plug was divided near the bottom, and the part attached to the abdominal wall was left behind. The parietal peritoneum surrounding the remnant mesh was exfoliated as in conventional transabdominal preperitoneal repair (TAPP). (C) After the parietal peritoneum was exfoliated, a mesh usually used for TAPP was inserted. The remnant of the previously placed mesh plug was covered by a newly placed mesh. (D) The newly placed mesh was covered with a peritoneal closure. White arrow, hernia defect; black arrow, previously placed mesh

**FIGURE 2:**
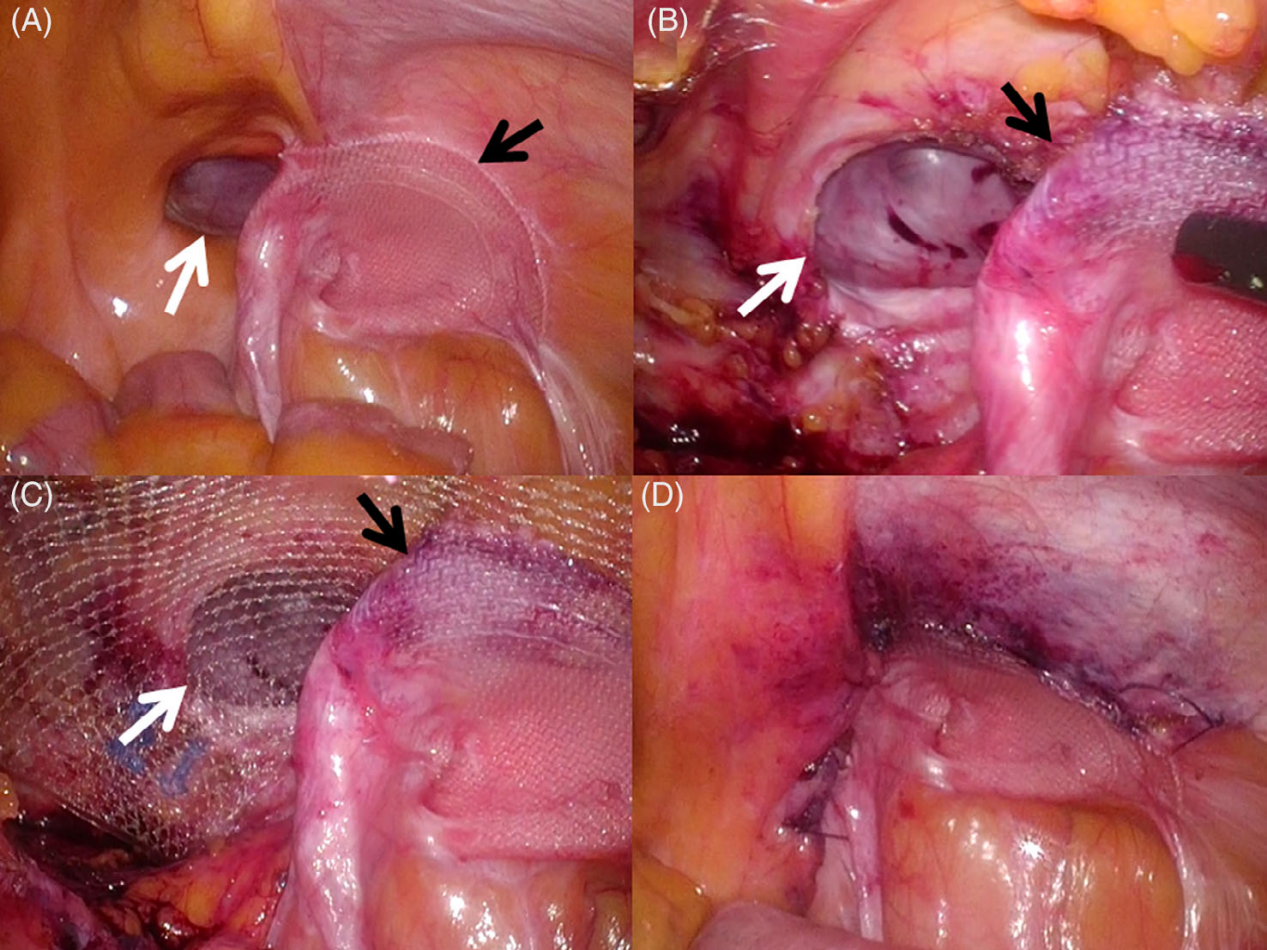
Intraoperative findings from modified transabdominal preperitoneal (TAPP) repair for right recurrent direct inguinal hernia that developed after transinguinal preperitoneal mesh repair. (A) The hernia defect was observed adjacent to the previously placed mesh, which was distorted and/or had shifted and no longer sufficiently covered the previous hernia defect. (B) The parietal peritoneum and the previously placed mesh over an area other than “trapezoid of disaster” were exfoliated. (C) Next, if the exfoliated peritoneum could sufficiently cover the newly placed mesh as a peritoneal closure, a mesh usually used for TAPP was inserted into the space in front of the urinary bladder. The myopectineal orifice was then covered by a combination of the previously and newly placed meshes. (D) The peritoneal incision was sutured and closed to cover the newly place mesh. White arrow, hernia defect; black arrow, previously placed mesh

**FIGURE 3:**
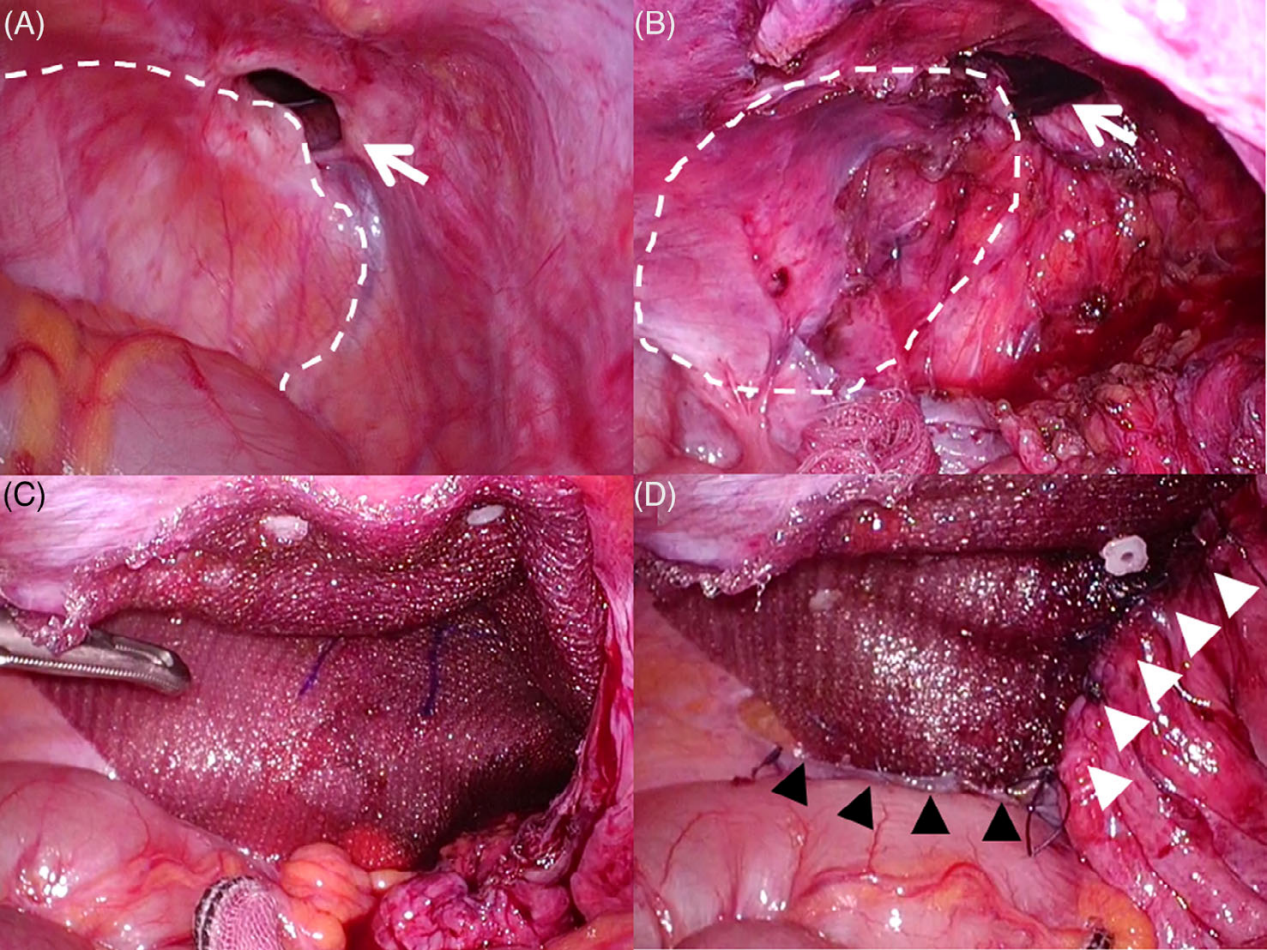
Modified intraperitoneal onlay mesh repair for left recurrent direct hernia that developed after transinguinal preperitoneal mesh repair. (A) The hernia defect was observed adjacent to the previously placed mesh. (B) In some cases, the exfoliated parietal peritoneum was insufficient to cover the newly placed mesh. When this occurred, a mesh coated with an absorbable hydrogel barrier for incisional hernia repair (Ventralight ST [VST]) was used. (C) The medial side of the VST was inserted into the space in front of the urinary bladder, and the portion above the iliopubic tract was fixed by tacking. The parietal peritoneum near the latero‐dorsal corner of the abdominal cavity was exfoliated slightly into the retroperitoneum to create a pocket to spread out the mesh to prevent it from rolling up. (D) After the ventral edge of the mesh was tacked above the iliopubic tract, the upper end of the peritoneum in the pocket (ie, the intra‐abdominal side of the pocket) was sutured to the surface of the VST. In other word's part of the mesh's dorsal edge was hidden in the pocket (black arrowheads). After that, the medial umbilical fold was sutured to a suitable site on the mesh's surface to hide the medial portion of the mesh in the space in front of the urinary bladder (white arrowheads). White arrow, hernia defect; white dotted line, previously placed mesh

Before each surgery, the formation pattern of the RIH defect was classified as either distant from the mesh (Figure 1) or adjacent to the mesh, which was distorted and/or had shifted and no longer sufficiently covered the previous hernia defect (Figures 2 and 3). The former was usually observed in cases of prior MPR and the latter in cases of prior TIPPMR.

In surgery for RIH distant from the mesh, we divided the previously placed mesh plug near the bottom and left the portion of the plug that was attached to the abdominal wall (Figure 1). After that, the parietal peritoneum surrounding the remaining portion of the plug was exfoliated. In other words, most cases of RIH after MPR could be repaired by the conventional TAPP procedure but without plug removal (Figure 1).

In surgery for RIH adjacent to the mesh, we first attempted to exfoliate the portion of the mesh covering the area other than the “trapezoid of disaster.” To prevent injury, we did not exfoliate the portion of the mesh covering the “trapezoid of disaster.” For this part of the mesh, we exfoliated the parietal peritoneum from the surface of the mesh if possible. We continued to exfoliate the parietal peritoneum around the Hesselbach triangle and subsequently entered the space between the urinary bladder (UB) and the pubic bone, ligament of Cooper, or rectus muscle to create sufficient space in front of the UB for placing new mesh.

Most cases of RIH after OPMR had a direct inguinal hernia, and the region around the internal inguinal ring was covered with the previously placed mesh. Therefore, space was created in front of the UB to enable subsequent mesh placement. In cases of RIH with an indirect inguinal hernia, exfoliation around the internal inguinal ring was necessary.

After the parietal peritoneum was exfoliated to create the space, a mesh usually used for TAPP (3D Max Light Mesh; C. R. BARD, Inc., Franklin Lakes, New Jersey) was placed if the exfoliated peritoneum could sufficiently cover the newly placed mesh as a peritoneal closure. The mesh was fixed to the abdominal wall with a tacker. The myopectineal orifice was then covered by a combination of the previously and newly placed meshes (Figure 2). We called this procedure the modified TAPP.

In cases in which the exfoliated peritoneum could not insufficiently cover the newly placed mesh, a mesh coated with an absorbable hydrogel barrier for incisional hernia repair (Ventralight ST; C. R. BARD, Inc.) (VST) was used (Figure 3). When the VST was placed, the medial side was inserted into the space in front of the UB and the portion above the iliopubic tract (IPT) was fixed by tacking. Additionally, the parietal peritoneum near the latero‐dorsal corner of the abdominal cavity was exfoliated slightly into the retroperitoneum to create a pocket to spread out the mesh to prevent its dorsal edge from rolling up. After the ventral edge of the mesh was tacked above the IPT, the upper end of the peritoneum in the pocket (ie, the intra‐abdominal side of the pocket) was sutured to the surface of the VST. In other words, part of the mesh's dorsal edge was hidden in the pocket (Figure 3). After that, the medial umbilical fold was sutured to a suitable site on the mesh's surface to hide the medial portion of the mesh in the space in front of the UB (Figure 3D). We called this procedure the modified intraperitoneal onlay mesh (IPOM).

## 3 RESULTS

Between April 2016 and December 2019, 11 RIH lesions in 10 patients were surgically treated by CLR. PAOR had been performed with TIPPMR in seven lesions and MPR in four. All 10 patients were men. Two patients had undergone PAOR at hospitals other than ours, and details of their previous surgery had been unknown until intraoperative laparoscopic findings indicated that PAOR had been performed. The median age at RIH surgery was 70 years (range, 42‐84 years). The median time from PAOR to the development of RIH was 1.3 years (range, 0.2‐15 years). The time to recurrence was determined based on when the patient indicated it developed. RIH was a direct inguinal hernia in 10 cases and an indirect inguinal hernia in 1 case.

To repair the RIH, the modified TAPP was employed in five cases, the modified IPOM in four, and the conventional TAPP in two. The median operative time was 113 minutes (range, 75‐170 minutes). (In the case with bilateral RIH, the total operative time was divided by two, and the quotient was used as operative time for each side of the RIH.) Two patients developed seroma as an early postoperative complication after modified IPOM and were conservatively cured. During the follow‐up period (range, 3‐45 months; median, 19 months), neither chronic pain nor re‐recurrence was observed in any patients.

## 4 DISCUSSION

To validate our current strategy for treating RIH after OPMR, further analysis involving many more cases and longer follow‐up period is needed. In the meantime, we will continue to perform CLR for RIH after OPMR because the results of this small case series were favorable.

## DISCLOSURE OF INTERESTS

The authors have no conflicts of interest to report and did not receiving any funding for this study.

## AUTHOR CONTRIBUTIONS

D.M., Y.I., M.M., and Y.S. had full access to all the study data and are responsible for the integrity of the data and the accuracy of the data analysis.

Study concept and design: D.M., K.Y., N.O., and Y.S.

Data acquisition: D.M., Y.I., N.O., K.Y., N.H., F.A., and Y.S.

Data analysis and interpretation: D.M. and Y.S.

Manuscript draft: D.M., Y.I., and Y.S.

Critical revision of the manuscript for important intellectual content: D.M., Y.I., K.Y., N.H., N.O., F.A., M.M., and Y.S.

Study supervision: Y.I., N.O., M.M., and Y.S.
